# Surgical Aortic Valve Replacement in Cancer Survivors with Severe Symptomatic Aortic Valve Disease: A Retrospective Single-Center Observational Study

**DOI:** 10.3390/cancers17203301

**Published:** 2025-10-12

**Authors:** Ivo Deblier, Ruben Deblier, Wilhelm Mistiaen

**Affiliations:** 1 Department Cardiovascular Surgery, ZNA Middelheim General Hospital, Lindendreef 1, 2020 Antwerp, Belgium; ivo.deblier@zas.be; 2 Faculty of Medicine and Health Sciences, University of Antwerp, 2610 Antwerp, Belgium; ruben.deblier@student.uantwerpen.be

**Keywords:** aortic valve replacement, malignancy, survival

## Abstract

**Simple Summary:**

Because of improved prognoses, cancer survivors with symptomatic aortic valve disease can be referred for cardiac surgery. However, the postoperative outcome of patients with this complex condition is uncertain. In a retrospective monocentric series totaling 2500 patients, 388 cancer survivors were compared with aortic valve patients without prior cancer. The referral increased over time, and cancer survivors were more often male and had more kidney disease but less pulmonary disease. Preoperative severity of heart disease, complexity of surgery, rate of adverse events, 30-day mortality, and need for resources were comparable. Long-term survival was significantly decreased by about one year and was driven mostly by an interval between cancer treatment and cardiac surgery of less than 5 years and lung cancer lethality. Prior malignancy was the least important of the ten identified predictors. For patients with lung cancer, the outcome is poor.

**Abstract:**

Background/Objectives: Cancer survivors can develop heart conditions such as aortic valve disease because of age and other shared risk factors. If this valve condition becomes symptomatic, the prognosis is poor if the valve is not replaced. Surgical aortic valve replacement (SAVR) is one mode of treatment. Methods: Of 2500 consecutive patients who underwent SAVR with a biological valve, 388 patients were cancer survivors. They were compared for preoperative characteristics, operative parameters, postoperative adverse events, need for resources, and long-term survival. For the latter, the six most common tumors (prostate, breast, colorectal, bladder, pulmonary, and hematologic) and the effect of the interval between cancer treatment and cardiac surgery were scrutinized. Results: Cancer increased significantly over time. Pulmonary and kidney disease differed between the groups, but cardiac comorbid conditions did not. Operative parameters, early adverse events, and need for resources did not differ. Median survival time was significantly reduced in cancer survivors: 104 (97–112) versus 119 (116–122) months, and this was driven by an interval of less than 5 years and prior lung cancer. Prior cancer was the least important of ten predictors for long-term mortality. Conclusions: The outcome of cancer survivors after SAVR is acceptable. For patients with pulmonary cancer, the outcome is poor.

## 1. Introduction

Malignancy and heart disease are the leading causes of death in Western societies [1,2,3,4]. There are several reasons why cancer survivors experience heart disease. These reasons include an increased life expectancy [5], improvement in the management of cancer [6], and common risk factors for both conditions. These include smoking, diabetes, obesity, and physical inactivity [2,7,8]. These risk factors were significantly more present in cancer survivors compared to matched controls [9]. Chronic inflammation and oxidative stress may have a considerable role in both cancer and cardiovascular disease [10,11]. Inflammation and oxidative stress are also involved in the development of calcific aortic valve stenosis [12]. Cancer treatment could also have an impact on the progression of aortic valve sclerosis by major non-cardiac surgery, by autologous stem cell transplantation [13,14,15], or by mediastinal radiotherapy [16,17,18,19,20]. Irradiation of valvular tissues leads to fibrosis with thickening of the leaflets, calcification, loss of mobility with initial regurgitation with progression to stenosis, and involvement of the aortic root and the mitral valve apparatus [21]. Despite the relationship between malignancy and heart disease, the incidence of prior cancer in cardiac patients varies. In our prior experience, this was 2.4% in a patient group referred mostly for coronary artery bypass graft (CABG) surgery [22] and 9.5% in patients referred for surgical aortic valve replacement (SAVR) [23]. The prevalence of prior malignancy in patients with severe aortic valve stenosis could be 20% or more in other series [16,19,24,25]. However, the incidental finding of an active malignancy in elderly patients referred for aortic valve replacement of any type was much lower, between 2.5 and 4.5% [26,27,28,29]. Both conditions limit life expectancy severely if left untreated. Congestive heart failure in aortic valve disease is a main driver for mortality in cancer patients [2,30]. Valve replacement resulted in improved survival compared to medical treatment [31]. In the past, cardiac surgeons were reluctant to perform operations on cancer survivors because of an anticipated lower survival. Encouraging short- and mid-term results were observed after cardiac surgery in these patients [32], and a prior malignancy was not identified as a predictor for 30-day mortality after SAVR [23]. An interval between cancer treatment and cardiac surgery of 2 years or less was the main driver of reduced long-term survival [22]. A survival benefit was observed in patients with severe aortic valve stenosis with simultaneous cancer if the latter was treated in an early stage [2]. Transcatheter aortic valve implantation (TAVI) has been introduced as a less invasive treatment for high-risk patients and for patients with an otherwise short life expectancy. Patients with an acceptable prognosis after oncologic treatment could benefit from valve replacement [16,19]. There are, however, very few series studying the outcome of cancer survivors after SAVR in the long-term, with results after 10 years or more. This is, to our knowledge, is the first large-scale monocentric series with respect to this complex problem. The research questions for patients referred for SAVR and prior malignancy are (1) What is the effect of the introduction of TAVI in 2008 on the referral of cancer survivors for SAVR? (2) Do patients with a prior malignancy have a higher degree of other comorbid conditions? (3) What is the early outcome (<30 days) after SAVR in these patients? (4) Do these patients need more postoperative resources? (5) What is the long-term outcome in these patients, especially after 10 years? (6) What is the long-term effect of the six most common malignancies? (7) What is the effect of the interval between the cancer treatment and SAVR on the outcome?

## 2. Materials and Methods

This is a retrospective study of 2500 consecutive patients who underwent SAVR in a general hospital from 1 January 1987 until 6 July 2017. Of these, 388 patients (15.5%) had a histologically documented and treated malignancy. Exclusion criteria were implantation of mechanical valve prostheses and biological valve prostheses in any other position. Patients with concomitant procedures such as CABG, mitral valve repair, and a procedure on the ascending aorta were not excluded. The definitions of demographics, preoperative comorbid conditions, severity of heart disease, operative characteristics, postoperative adverse events, and need for resources were defined in prior papers [33,34]. Patients were considered to have a previously treated malignancy if this was histologically documented during prior admissions or if they had a curable active malignancy. A chi-square analysis was used to identify associations of preoperative, operative, and postoperative factors with malignancy for categorical variables. For continuous variables, a Student’s t-test was used. The effect of prior malignancy on long-term survival was investigated with a log-rank test. To rank the factor malignancy amongst other predictors for survival, a multivariate Cox’ proportional hazard analysis was used. The effect of the time interval on survival was determined by a univariate Kaplan-Meier analysis.

## 3. Results

### 3.1. Preoperative and Operative Characteristics

**Figure 1:**
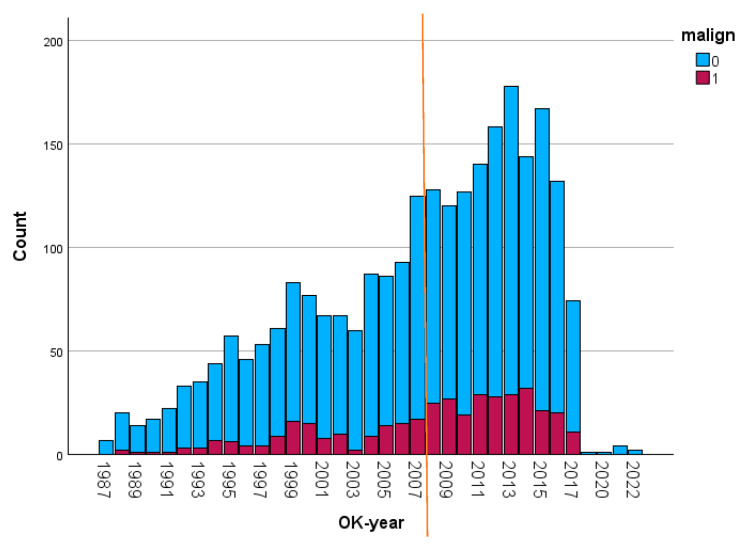
Annual number of patients referred for SAVR (the darker bars represent the patients with a prior malignancy). Malign: malignancy; OK-jaar: year of operation. Referral pattern of patients over time.

Even after the introduction of TAVI in 2008 (indicated by the line in Figure 1), the number of referrals of cancer survivors (dark color) for SAVR continued to increase. The inclusion stopped in July 2017, explaining the lower numbers at the right side of the figure.

Table 1 shows the distribution of malignancies. As in the general population, prostate, breast, and colorectal carcinomas were most frequent. Some malignancies, such as cerebral tumors, were absent because of their poor prognosis. Hematological malignancies included all types of leukemias, lymphomas, and myelodysplastic syndromes. In ten patients, more than one prior malignancy was diagnosed.

Table 2 shows the association of preoperative and operative factors with malignancy. Patients with prior malignancy were significantly more referred after 2008. Male gender, chronic kidney disease (defined as glomerular filtration rate <60 mL/min), prior pacemaker implant, and cerebrovascular accident were more present in patients with prior malignancy. Obesity, hyperlipidemia, and pulmonary dysfunction were less present in patients with prior malignancy. Aortic valve area (73 ± 26 mm^2^ vs. 74 ± 30 mm^2^, *p* = 0.659), LV ejection fraction (61 ± 16% vs. 62 ± 10%, *p* = 0.437), and Euroscore II (6.7 ± 7.5% vs. 6.7 ± 7.9%, *p* = 0.937) were comparable for both groups. BMI was significantly lower in cancer patients (27.2+/−9.2 vs. 26.1+/−3.8, *p* = 0.005), while age was higher (75.2+/−6.6 vs. 76.2 +/−5.9 years, *p* = 0.004). The association with operative characteristics showed no differences between the groups. The cardiopulmonary bypass time did not differ between these groups (118.2 ± 40.7 vs. 122.4 ± 44.3, *p* = 0.193). Cross-clamp time was 3 min shorter in the cancer group: 66.1 ± 19.6 vs. 69.2 ± 23.0 min. This was significant (*p* = 0.023), but a low effect size of 0.137.

### 3.2. Early Postoperative Outcome

Table 3 shows the absence of significant differences in postoperative events between patients with and without malignancy, except for atrial fibrillation, which was less present in patients with prior malignancy. Hospital mortality, as the most important short-term outcome, was 107/2139 (5.0%) for patients without a prior malignancy and 27/388 (7.0%) for patients with malignancy, which is not significantly higher (*p* = 0.114). For patients with prior malignancy, the Euroscore predicted very well the hospital mortality. Forty patients had a hematologic malignancy, with four mortalities, one due to an infection because of a recently discovered large-cell lymphoma. Six patients were under treatment for chronic lymphocytic leukemia. Three of them had severe complications (need for reintervention, shock with secondary infection, atelectasis), but without mortality. Mechanical ventilation was 6 h longer in cancer survivors (18.0 ± 56.9 vs. 12.1 ± 36.4, *p* = 0.112). The length of stay in the intensive care unit (3.1 ± 7.2 days vs. 3.4 ± 9.0 days, *p* = 0.483) and postoperative length of stay (10.4 ± 8.7 days vs. 10.0 ± 7.5 days, *p* = 0.445) differed hardly. Postoperative hematocrit values (24.9 ± 3.5% vs. 24.7 ± 3.0%, *p* = 0.281) and the number of administered units of packed cells (2.7 ± 3.5 vs. 2.9 ± 3.6, *p* = 0.279) were also not different.

Table 4 shows the effect of the six most common malignancies on 30-day mortality. Only prior pulmonary cancer had a significant effect on this outcome, although the numbers were low. The *p*-values refer to the difference with patients without prior malignancy.

### 3.3. Long-Term Postoperative Results

**Figure 2:**
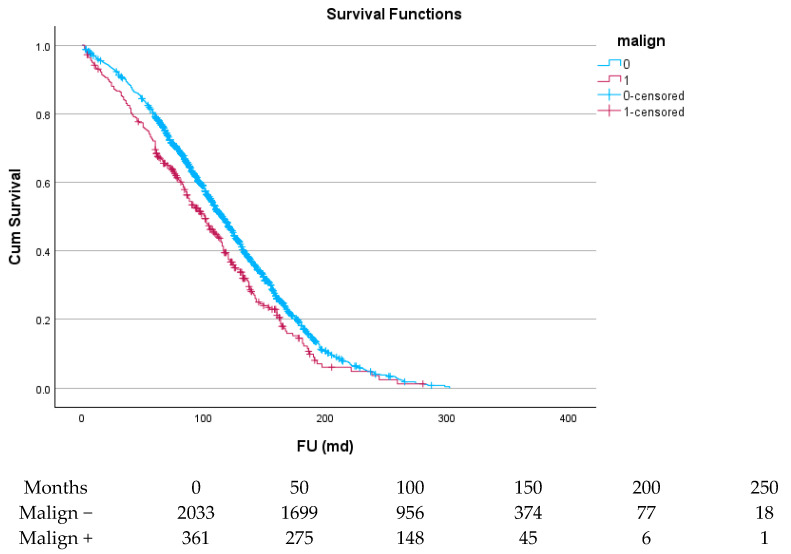
Survival curve of patients with malignancy versus patients without malignancy.

There was a total of 21,194 patient-years for long-term follow-up. For cancer patients, this was 2858 patient-years. As can be derived from Figure 2, the 1-year survival for all cancer patients differed little between both groups. After some divergence, the curves ran parallel for most of the duration of the follow up. Median survival time in patients without prior malignancy was 119 (116–122) months. For patients with malignancy, this was 104 (97–112) months, with *p* < 0.001. One-, five-, ten-, and fifteen-year survival for patients without prior malignancy were 96.0+/−0.4%, 79.2+/−0.9%, 47.1+/−1.2%, and 18.2+/−0.11%. For patients with malignancy, this was 93.1+/−1.3%, 69.6+/−2.4%, 37.5+/−2.8%, and 13.0+/−2.5%, respectively. In patients undergoing isolated SAVR, 5-year survival was 81.3+/−3.4% (without malignancy) vs. 72.3+/−3.8% (with malignancy), while in patients with a complex procedure, 5 year survival was 77.9+/−1.2% (without malignancy) vs. 68.3+/3.1% (with malignancy). For both patient groups, the reduction in survival because of a malignancy was 9%. In the era before 2008, 5-year survival was 79.1+/−1.3% (without malignancy) vs. 66.1+/−3.7% (with malignancy). In the era after 2008, 5-year survival was 79.2+/−1.3 (without malignancy) v. 73.1+/−3.2 (with malignancy). The differences in outcome between patients with versus without malignancy narrowed over time.

**Figure 3:**
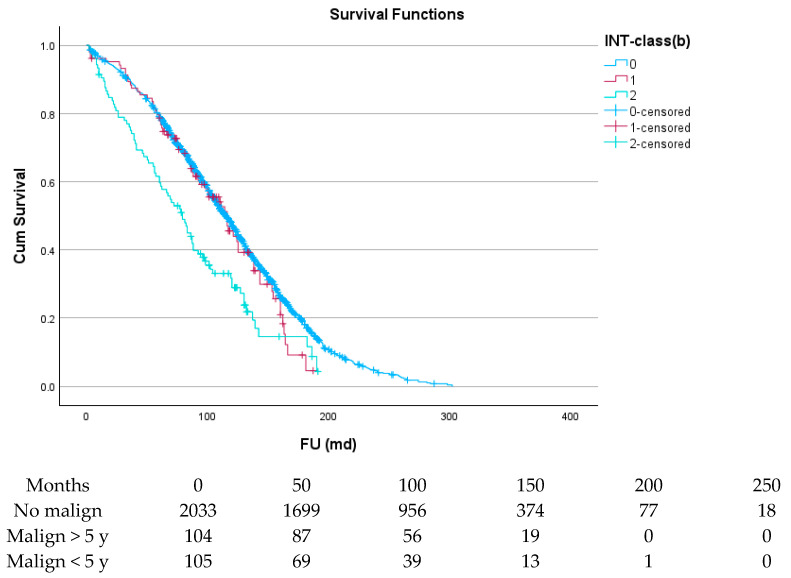
The effect of interval between malignancy and SAVR on survival.

Figure 3 and Table 5 show the effect of the interval between cancer and cardiac surgery: the survival of patients with an interval of 5 years or more (green line) is almost identical to that of patients without prior malignancy (blue line), while for patients with an interval of less than 5 years (red line), this was significantly less (*p* < 0.001).

**Figure 4:**
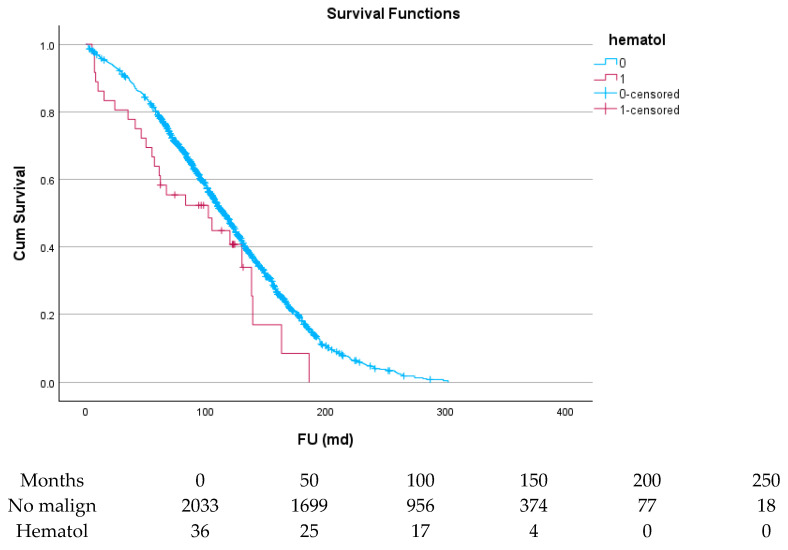
Effect of hematologic malignancy on survival.

**Figure 5:**
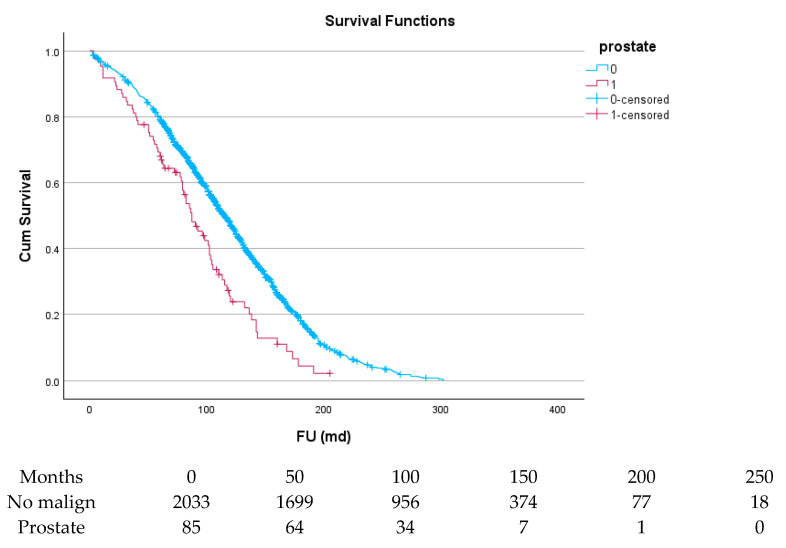
Effect of prostate cancer on survival.

**Figure 6:**
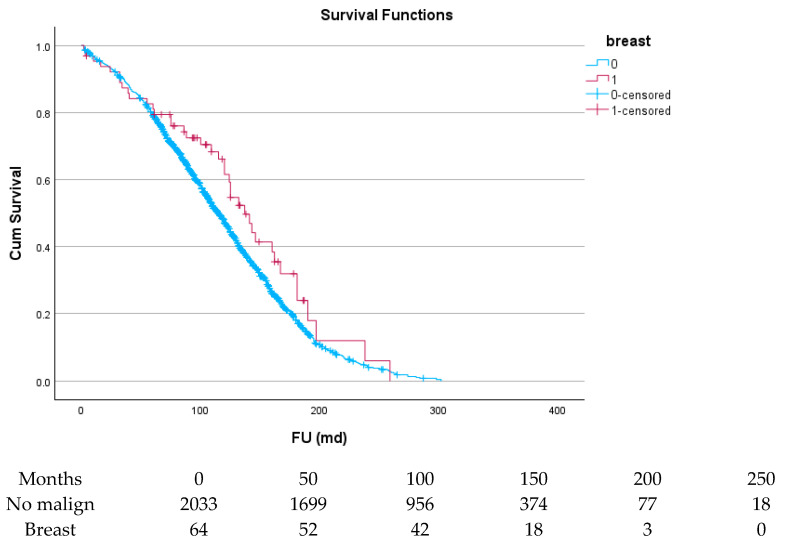
Effect of breast cancer on survival.

**Figure 7:**
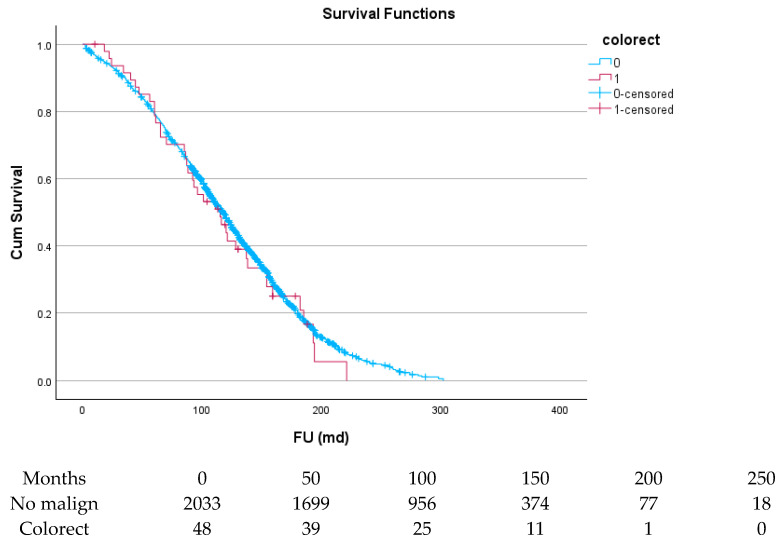
Effect of colorectal cancer on survival.

**Figure 8:**
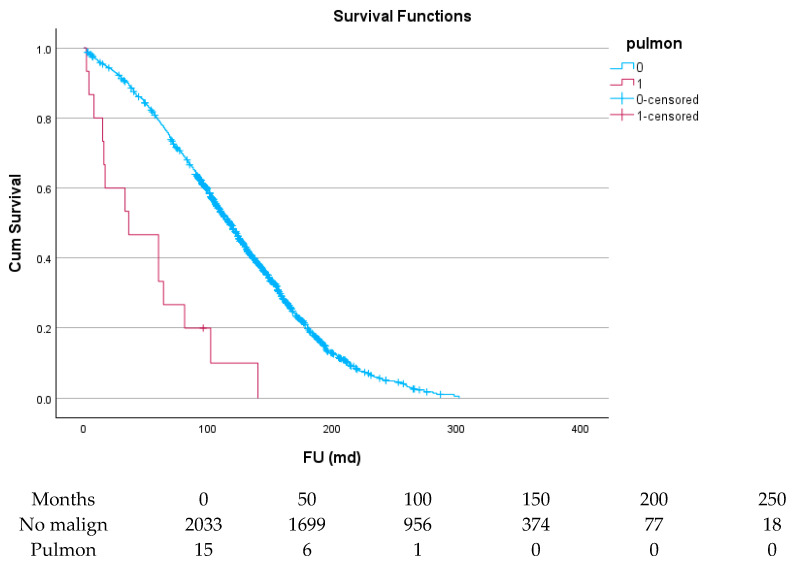
Effect of pulmonary cancer on survival.

**Figure 9:**
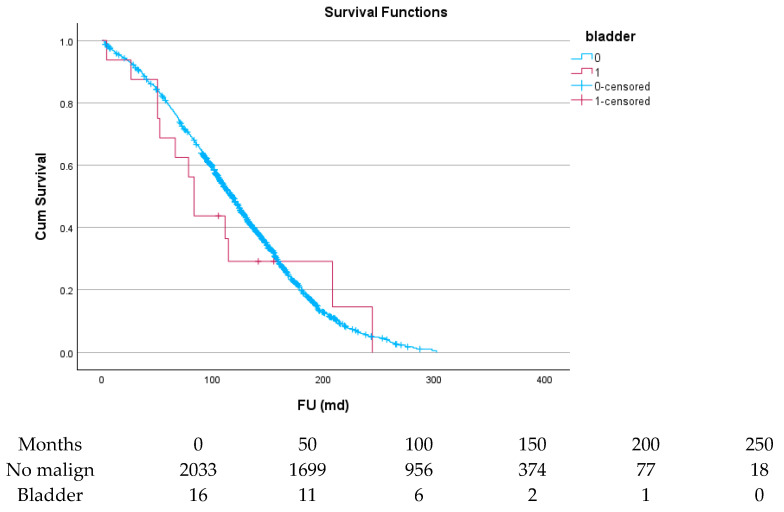
Effect of bladder cancer on survival.

Table 6 and Figure 4, Figure 5, Figure 6, Figure 7, Figure 8 and Figure 9 show the long-term survival for the different groups of patients. This corresponds with the curves. For patients with hematologic malignancy, pulmonary, and prostate cancer, survival was significantly reduced. For the other types of cancer, the difference in survival was not significant. The difference in survival was especially low in patients with pulmonary tumors.

Table 7 shows the ten independent predictors for long-term mortality. Age over 80 is the most significant and clinically relevant predictor in terms of odds ratio. Other comorbid conditions such as peripheral artery disease, high New York Heart Association (NYHA) functional class, and chronic renal and pulmonary disease are also highly significant, albeit with a lower odds ratio. Malignancy ranks lowest according to the *p*-value and has a relatively low odds ratio.

In 128/388 patients (32.9%) with prior malignancy, there was either a new malignancy or a progression of the preoperative malignancy. In 52 (13.4%) patients, this was a new malignancy. In the patients with a documented progress of prior malignancy, the interval was mostly less than 2 years. In 282/2143 (13.1%) patients without prior malignancy, a postoperative malignancy was documented during follow-up. This was similar to a second, unrelated malignancy observed in patients with prior malignancies (*p* = 0.920). A new postoperative cancer or progression of a prior malignancy resulted in a reduced 5-year survival from 82.4+/−1.1% to 74.0+/−2.2% (*p* < 0.001).

## 4. Discussion

This is one of the few large monocentric series dedicated to the difficult problem of cancer survivors needing SAVR. Its duration of follow-up is also exceptionally long. It also studies the effect of the six most common malignancies and the effect of the time-interval between malignancy and cardiac surgery. This interval serves as a chance for a cure. Our results show that patients with a prior or currently treatable malignancy have a comparable short-term outcome and need for postoperative resources. The long-term survival is reduced by 15 months. This reduction is mainly driven by a time interval between cancer treatment and cardiac surgery of less than 5 years. The presence of certain malignancies, such as lung cancer, and, to a lesser degree, prostate cancer and hematologic malignancies, also results in a more pronounced decreased survival rate. There is a reasonable expectation that the patient will experience long-term clinical improvement and increased life expectancy after SAVR. The prognosis of malignancy may limit the selection of patients for cardiac surgery, but this could also apply for other conditions such as severe chronic pulmonary or renal disease. Valve disease has been acknowledged as a serious side effect of cancer treatment such as radiotherapy of the chest. Because of radiation therapy, endothelial cells thicken and could become dysfunctional. The valve tissue starts to calcify [19]. In prior series, patients with prior radiotherapy occurred in an important part of the patients referred to SAVR [35]. Despite an increasing need for aortic valve replacement in cancer survivors, patients often were not considered for surgical treatment [19]. The incidence of prior cancer in our series increased significantly over time, from 11.4% before the introduction of TAVI in 2008 to 17.7% thereafter. In a prior series, this cancer incidence increased with patient age and was comparable to current levels [36] but lower than the observed 22.6% in those who underwent TAVI [19]. This rate of cancer is higher compared to the series of cardiac surgery in general, which varies between 2% and 8% [22,35,37,38].

In the current series, patients with a prior malignancy were significantly older compared to those without cancer, but this difference was limited to one year. This difference was larger in a previously published nationwide series for patients undergoing SAVR: 69 versus 73 years. Patients referred to TAVI had a mean age of 81 years, irrespective of their cancer status [19]. In another series, the mean age of cancer patients with aortic valve disease was 70 years for medical treatment, 72 years for TAVI, and 74 years for SAVR. This group was relatively small [3]. In the current series, prior permanent pacemaker implant, chronic kidney disease, prior stroke, and male gender were more present in cancer survivors. These patients had less hyperlipidemia and chronic pulmonary disease. Other cardiovascular factors and operative data showed no significant difference. In previously published series, cancer survivors referred for SAVR had a considerably higher rate of coronary artery disease, prior percutaneous coronary interventions, atrial fibrillation, arterial hypertension, hyperlipemia, coagulation disorders, prior stroke, chronic kidney and pulmonary dysfunction, and smoking habits, but less obesity and cardiomyopathies. For diabetes, prior myocardial infarction, vascular disease, and neuropsychiatric disorders, there was no difference between cancer survivors and non-cancer patients [19]. Patients who underwent prior radiotherapy of the chest had more LAD disease on top of aortic valve stenosis, but the aortic valve disease was less severe. More bypasses were needed after chest radiotherapy [17]. Prior endocarditis was rare in the current series. The existence of a carcinoma can be associated with endocarditis through a port of entry such as colorectal neoplasias and immunosuppressive cancer treatments. If a cancer is treatable, valve surgery should not be withheld in these severely ill patients [39,40]. As in our series, prostate, breast, and colon cancers were most common in patients referred for aortic valve replacement [19,37,38]. In another Japanese series, the most common tumors were prostate, lung, and gastric cancer [41]. Hematologic malignancies were observed in 11% to 17% of the cancer survivors [35,38] and in 31% in another small series [3].

In the current patient series, the mortality rate was 5% for patients without a malignancy and 7% for patients with a malignancy, which was close to the observed Euroscore of 6.70%. Except for atrial fibrillation, there were no more postoperative events in the current group of cancer survivors. The need for resources was also comparable. In prior series, a low hospital mortality after cardiac surgery was also observed in cancer survivors [32,37,38]. One specific subgroup consisted of patients with prior chest radiotherapy. These patients suffered more from stroke, atrial fibrillation, and mortality after SAVR [17]. About 10% of the current patients with a malignancy had a hematologic disease. These patients had a higher mortality rate of 10%, but this difference did not reach statistical significance because of the low numbers. The morbidity rate was significant, however. Bleeding and infections were most important [42,43,44], but an increase in pulmonary and renal complications has also been observed [45]. The increased need for resources was closely related to postoperative complications. The need for associated operative procedures and postoperative resources in patients with malignancy was not higher in the current series. This was reflected by a comparable cardiopulmonary bypass time and need for blood products, prolonged ventilation or renal replacement therapy, or pacemaker implant. The length of stay in the ICU or in the hospital ward was also comparable. A similar observation was made not only for patients with past but also with active malignancies [37]. However, in several series, the need for intra- or postoperative transfusions was higher in patients with lymphoma or leukemia [35,43,44,45,46]. Reoperation rate for various reasons was also high in patients with hematologic malignancies [42]. The length of stay in the intensive care unit was prolonged to a limited degree [45]. Patients with prior chest RT had increased surgical times, an increased need for blood products and permanent pacemaker implantation, as well as a prolonged length of stay in the intensive care unit. Readmission rate at three months postoperative was higher [17].

Long-term survival in the current series was significantly lower for cancer survivors and was driven by a shorter interval between cancer treatment and SAVR and by the type of malignancy. Current results showed a divergence in survival curves after one year: the difference in 1-year survival was about 2%. After 5 and 10 years, this was almost 10%. Previously observed median survival time for patients with prior hematologic malignancy was approximately halved from 12 to about 6.5 years [45]. Radiotherapy in patients with prior lymphomas can be associated with an increased rate of organ related complications, but the postoperative long-term results still were acceptable [46]. Lower survival after SAVR, in general, was mainly attributed to cardiovascular events. This pointed to the need for earlier intervention but also to the need for a closer postoperative follow-up [47]. Although prior malignancy was currently identified as an independent predictor for long-term survival, it ranked only 8th out of 10 parameters for its significance, while age, renal and pulmonary dysfunction, as well as peripheral artery disease, and diabetes were more important. In terms of clinical relevancy, expressed as an odds ratio, malignancy ranked as the last of ten predictors. A second malignancy did not occur more often with a first malignancy during long-term follow-up compared to a first malignancy in patients without such history. A lower survival rate because of metastasis could be expected if the interval between malignancy and cardiac surgery was less than 5 years. With a smaller interval, the chance of cure could be considered less likely [22]. Readmission for malignancy and cancer mortality were higher in patients with active cancer, i.e., no interval [37]. One-year survival after cardiac surgery (mostly CABG) is not affected by the interval of 5 years [35]. The stage of cancer also showed an effect on survival in prior series, with a higher survival in early stages, but even in patients with active cancer and severe aortic valve stenosis, a survival benefit of SAVR could be documented [48].

The current results show that the introduction of TAVI as a less invasive treatment for aortic valve stenosis did not result in a diminished referral of patients for SAVR. Earlier series indicated that reasons to replace the valve in cancer survivors were the severity of valve symptoms, a requirement to replace the valve in anticipation of cancer surgery, and the severity of aortic valve stenosis, while absence of symptoms and a limited life expectancy not related to the valve condition were reasons not to replace the valve by either method [41]. TAVI has the advantage of being less invasive and allows earlier resumption of anti-cancer treatments, without having a difference in overall survival between SAVR and TAVI. Moreover, the introduction of TAVI could reduce the number of patients who would otherwise not be considered for treatment of valve disease [1,49,50]. Despite the introduction of TAVI in 2008, we documented an increase in referrals for SAVR. The ratio of patients with cancer also increased over time. We observed a difference in postoperative 5-year survival before 2008 of about 13%, while after 2008, this was about 6%. The difference in survival between cancer and no-cancer patients clearly narrowed due to improvement in care.

This study has clear limitations. TAVI, as a recognized treatment of aortic valve disease, was not a part of the study design. There is a long inclusion time with changing operative and perioperative care and oncologic treatment modalities. Current data, however, do not allow us to make a distinction between the improvements in cancer treatment and in cardiosurgical techniques over time. Other limitations are the lack of information about the stage of cancer and the treatment modalities, but treatment with curative intent or a potential cure of concurrent malignancy can be assumed. The interval between cancer treatment and SAVR can be considered as a measure for the likelihood of cure. This parameter is known in most patients. The current follow-up with respect to mortality is complete, but the cause of death is not always known. The reason is the admittance in skilled nursing facilities of many patients because of high age and other, unrelated disabilities such as dementia and poor mobility. An analysis for competing causes of death is therefore unreliable. The low survival in patients with a postoperative progression of a prior malignancy is an indication of the importance of the interval between the malignancy and cardiac surgery. The follow-up is through exploration of the digital files and depends on the completeness of the information. There is limited availability about past or current smoking and other relevant habits concerning cancer. Both patient groups had some differences in preoperative comorbid conditions. A Cox’ proportional hazard analysis was used as a valid alternative for a propensity score match analysis.

## 5. Conclusions

In conclusion, patients with a history of malignancy are older and have more chronic kidney disease but less pulmonary dysfunction compared to their counterparts without malignancy. Euroscore II, however, is not affected by the presence of malignancy, and cardiovascular characteristics are not significantly different. The complexity of surgery and the need for resources are also comparable, as well as short-term outcomes. The reduction in postoperative long-term survival in patients with malignancy is significant and is driven by an interval of less than 5 years between cancer treatment and SAVR and by specific types of malignancies, such as lung cancer. However, malignancy itself is one of the least important independent predictors for this outcome. Every effort should be made by a cardio-oncologic team to select these patients that will benefit from valve replacement. The introduction of TAVI did not reduce the referral of cancer survivors with severe aortic valve disease for SAVR. Surgery remains a viable treatment option for these patients.

## Figures and Tables

**Table 1:** Types of malignancies.

Prostate	97 *
Breast	68 *
Colorectal	54 *
Hematologic	40
Lung	18
Bladder	16
Kidney	12
Melanoma	7 *
Larynx	6
Cervix and endometrium	6
Esophagus	5
Gastric	4
Thyroid	4
Other	63
Total	388

* Includes patients with >1 malignancy.

**Table 2:** Distribution of preoperative and operative factors across patients with and without malignancy.

Factor	Malignancy + (%)	Malignancy − (%)	*p*
Preoperative factors			
Era after 2008	214/388 (55.2)	1035/2141 (48.3)	0.014
Prior PPM implant	24/387 (6.2)	77/2140 (3.6)	0.016
Chronic kidney disease	79/388 (20.4)	335/2134 (15.7)	0.023
Cerebrovascular accident	51/388 (13.1)	202/2135 (9.5)	0.026
Male gender	241/388 (62.1)	1205/2141 (56.3)	0.033
Hyperlipidemia	145/258 (56.2)	790/1255 (62.9)	0.042
COPD	261/1786 (14.6)	119/664 (17.9)	0.044
BMI > 30 kg/m^2^	60/258 (23.3)	350/1254 (27.9)	0.126
Age > 80 year	115/388 (29.6)	561/2141 (26.2)	0.159
Prior CABG	39/388 (10.1)	172/2141 (8.0)	0.186
Arterial hypertension	269/388 (69.3)	1543/2134 (72.3)	0.231
Congestive heart failure	107/387 (27.6)	532/2139 (24.9)	0.247
Coronary artery disease	235/388 (60.6)	1346/2141 (62.9)	0.389
Myocardial infarction	66/388 (17.0)	331/2135 (15.5)	0.453
Peripheral artery disease	126/344 (36.6)	692/1794 (38.6)	0.497
Endocarditis	12/388 (3.1)	54/2141 (2.5)	0.517
Diabetes mellitus	86/388 (22.2)	445/2141 (20.8)	0.539
Conduction defects	116/386 (30.1%)	668/2139 (31.2%)	0.645
SAVR < 24 h	22/388 (5.7)	111/2140 (5.2)	0.695
Atrial fibrillation	94/386 (24.4)	506/2141 (23.6)	0.760
Prior SAVR	11/388 (2.8)	56/2140 (2.6)	0.806
Prior PCI	41/388 (10.6)	226/2141 (10.6)	0.995
Operative factors			
Concomitant CABG	222/388 (57.2)	1292/2141 (60.3)	0.247
Concomitant CEA	6/388 (1.5)	49/2141 (2.3)	0.356
Ascending aorta	28/387 (7.2)	169/2140 (7.9)	0.655
CPB time > 120 min	130/340 (38.2)	674/1774 (38.0)	0.933
Mitral valve repair	15/388 (3.9)	83/2141 (3.9)	0.992

BMI: body mass index; CABG: coronary artery bypass grafting; CPB: cardiopulmonary bypass; PCI: percutaneous coronary intervention; PPM: permanent pacemaker; SAVR: surgical aortic valve replacement.

**Table 3:** Distribution of postoperative adverse events and need for resources across patients with and without malignancy.

Preoperative Factor	Malignancy + (%)	Malignancy − (%)	*p*
Categorical need for resources			
Postoperative LOS > 8 d	122/344 (35.5)	697/1791 (38.9)	0.228
>4 Units packed cells	60/259 (23.2)	258/1251 (20.6)	0.361
PPM implant	8/387 (2.1)	61/2137 (2.9)	0.382
Mechanical ventilation > 8 h	96/259 (37.1)	490/1249 (39.2)	0.515
Thrombocyte concentrate	29/259 (11.2)	156/1249 (12.5)	0.546
CVVH	17/387 (4.4)	81/2129 (3.8)	0.582
LOS-ICU	102/302 (33.8)	506/1560 (32.4)	0.650
Plasma products	76/259 (29.3)	356/1248 (28.5)	0.791
Reintervention	13/388 (3.4)	71/2141 (3.3)	0.972
Categorical adverse events			
Atrial fibrillation	134/387 (34.6)	858/2138 (40.1)	0.041
Mortality	27/388 (7.0)	107/2139 (5.0)	0.114
Ventricular arrhythmias	20/287 (5.2)	76/2136 (3.6)	0.128
Conduction defects	72/387 (18.6)	345/2137 (16.1)	0.230
Thromboembolism	17/387 (4.4)	71/2137 (3.3)	0.291
LCOS	32/387 (8.3)	147/2136 (6.9)	0.328
Low thrombocytes	32/262 (12.2)	183/1269 (14.4)	0.349
Delirium	42/347 (12.1)	186/1772 (10.5)	0.377
Bleeding events	27/387 (7.0)	128/2137 (6.0)	0.457
Pulmonary complication	45/388 (11.6)	289/2139 (13.5)	0.306
Acute renal injury	74/388 (19.1)	393/2136 (18.4)	0.753
Myocardial infarction	4/388 (1.0)	21/2139 (1.0)	0.928
Endocarditis	1/387 (0.3)	5/2137 (0.2)	0.928

CVVH: continuous veno-venous hemofiltration; complic: complication; ICU: intensive care unit; LCOS: low cardiac output syndrome; LOS: length of stay; LOS-ICU: length of stay in the intensive care unit; PPM: permanent pacemaker.

**Table 4:** Effect of different types of malignancy on 30-day mortality.

Factor	Present (%)	*p*
Colorectal	4/52 (7.5)	0.418
Hematological	4/40 (10.0)	0.174
Prostate	8/93 (8.6)	0.148
Breast	2/66 (3.0)	0.434
Pulmonary	3/18 (16.7)	0.030
Bladder	0/16 (0.0)	0.351

**Table 5:** Effect of the interval of 5 years on long-term survival (as % with 95% confidence interval).

Survival	No Malignancy	Interval > 5-Year	Interval < 5-Year
1-year	95.9 ± 0.4	96.2 ± 0.9	90.5 ± 2.9
2-year	93.2 ± 0.6	95.2 ± 2.1	80.8 ± 3.9
5-year	79.1 ± 0.9	78.7 ± 4.0	59.7 ± 4.8
10-year	47.1 ± 1.2	44.0 ± 5.5	29.0 ± 4.7
15-year	18.1 ± 1.1	9.2 ± 4.6	11.7 ± 4.6

**Table 6:** Survival analysis for the six most common types of malignancies (as % with 95% confidence interval).

	1-Year	5-Year	10-Year	15-Year	*p*
No malignancy	95.9 ± 0.4	79.1 ± 0.6	47.1 ± 1.2	18.1 ± 1.1	
All malignancies	93.1 ± 1.3	69.8 ± 2.4	37.9 ± 2.6	14.7 ± 2.3	<0.001
Pulmonary	73.3 ± 11.4	33.3 ± 12.2	10.0 ± 8.8	none	<0.001
Prostate	91.8 ± 3.0	68.1 ± 5.1	27.4 ± 5.1	6.6 ± 5.3	<0.001
Hematologic	83.3 ± 6.2	61.1+/_8.1	40.7 ± 8.3	13.6 ± 7.5	0.047
Breast	95.3 ± 2.7	81.0 ± 4.9	62.6 ± 6.2	31.2 ± 7.1	0.070
Colorectal	97.9 ± 2.1	78.7 ± 6.0	43.9 ± 7.3	20.0 ± 6.9	0.577
Bladder	90.0 ± 7.1	65.6 ± 11.8	29.2 ± 11.8	14.6 ± 11.9	0.581

**Table 7:** Independent predictors for long-term mortality.

Factor	OR	95% CI	*p*
Age > 80 years	2.30	1.95–2.71	<0.001
Peripheral artery disease	1.57	1.30–1.89	<0.001
Preop. NYHA functional class III/IV	1.50	1.26–1.79	<0.001
Chronic kidney disease	1.49	1.23–1.81	<0.001
Chronic obstructive pulmonary disease	1.43	1.22–1.67	<0.001
Incomplete revascularization	1.34	1.06–1.70	0.016
LV ejection fraction < 50%	1.19	1.03–1.38	0.020
Atrial fibrillation	1.22	1.02–1.45	0.031
Conduction defects	1.19	1.02–1.39	0.031
Malignancy	1.24	1.01–1.51	0.038

CI: confidence interval; LV: left ventricular; Preop. NYHA: preoperative New York Heart Association; OR: odds ratio.

## Data Availability

These results have been derived from a multipurpose database, from which several more publications will be derived. These data are not yet publicly available.
